# The Effectiveness of a Web-Based Application for a Balanced Diet and Healthy Weight Among Indonesian Pregnant Women: Randomized Controlled Trial

**DOI:** 10.2196/38378

**Published:** 2023-01-30

**Authors:** Mira Trisyani Koeryaman, Saseendran Pallikadavath, Isobel Helen Ryder, Ngianga Kandala

**Affiliations:** 1 Maternity Nursing Department Universitas Padjadjaran Jatinangor Indonesia; 2 Demography and Global Health School of Health and Care Professions University of Portsmouth Portsmouth United Kingdom; 3 Faculty of Nursing School of Health and Care Professions University of Portsmouth Portsmouth United Kingdom; 4 Faculty of Science School of Health and Care Professions University of Portsmouth Portsmouth United Kingdom

**Keywords:** web-based application, nutrition, pregnant women, pregnancy, mobile app, diet, dietary intake, application, Indonesia, randomized controlled trial, tool, consumption, maternal, weight, weight gain, intervention, control group, treatment, vitamins, minerals, healthy diet, calorie, food

## Abstract

**Background:**

Pregnant women have self-declared that they have difficulties in estimating nutrient intakes. The Nutrition Information System for Indonesian Pregnant Women (SISFORNUTRIMIL) application was created as a dietary assessment and calorie-counting tool to guide pregnant women to eat the right portion sizes for each meal.

**Objective:**

The study aimed to examine the effectiveness of the SISFORNUTRIMIL application in helping users achieve a balanced diet and healthy maternal weight gain in comparison to nonusers in Indonesia.

**Methods:**

First-pregnancy women in the second trimester aged 19-30 years (N=112) participated in the randomized controlled trial. Recruited women who were eligible and consented to participate in the study were allocated into the intervention group, or application user (n=56), and the control group, or application nonuser (n=56). The intervention recommended that pregnant women consume at least 5 food groups and calculate a recommended average portion size for 12 weeks. Both groups were self-monitored and recorded their intake in food records for 3 days every week. The dietary diversity consumed, macro- and micronutrient intake, and maternal weight gain were assessed pre- and postintervention. Data were collected three times during the intervention. Diversity food consumption was measured by the Minimum Dietary Diversity for Women of reproductive age. Furthermore, the Indonesian recommended dietary allowances were used to refer to and validate appropriate energy and nutrient amounts. Independent sample *t* test was used to compare differences between the intervention and control groups.

**Results:**

The mean dietary diversity score for the application user group (7.79, SD 1.20) was significantly greater than for the application nonuser group (7.02, SD 1.39; adjusted mean difference 0.77, 95% CI 0.28-1.25; *d*=0.28; *P*=.005). Macro- and micronutrient intake was significantly more in accordance with the dietary recommendations for the user group compared to the control group, including an energy daily intake of 156.88 kcal (95% CI 114.52-199.23; *d*=–1.39; *P*=.002), 102.43 g of carbohydrates (95% CI –125.2 to –79.60; *d*=–1.68; *P*=.02), 14.33 g of protein (95% CI 11.40-17.25; *d*=1.86; *P*<.001), and 10.96 g of fat (95% CI –13.71 to –8.20; *d*=–1.49; *P*<.001). Furthermore, there was a significantly higher intake of daily vitamins and minerals in the intervention group than in the control group. Other results showed that maternal weight gain in the intervention group was in accordance with the parameters of healthy weight gain.

**Conclusions:**

Recording food intake using the application was significantly effective in improving the dietary diversity consumed, improving adequate energy and nutrient intake, and producing healthy maternal weight during pregnancy.

**Trial Registration:**

ISRCTN Registry ISRCTN42690828; https://www.isrctn.com/ISRCTN42690828

## Introduction

### Background

An improved dietary diversity and diet quality during pregnancy are crucial for a healthy pregnancy and offspring, especially in low- and middle-income countries (LMIC) like Indonesia. Indonesia has persistently high maternal mortality and childhood stunting caused by inadequate maternal and child nutrition [1]. The Indonesia Demographic and Health Survey in 2017 reported that the prevalence of anemia and chronic energy malnutrition in pregnant women was 17.3% and 37.1%, respectively [2]. This data indicates adverse nutrition practices among pregnant women in Indonesia. Many factors could affect quality dietary intake during pregnancy, including affordability and accessibility [2,3]. Pregnant women have been prohibited from eating certain foods in some communities in Indonesia due to beliefs and food taboos. Numerous studies conducted in Indonesia have researched cultural practices and poverty that have caused undernourishment in pregnant women. The results have indicated that pregnant women in several regions of Indonesia avoid consuming particular foods such as cassava, durian, eggplant, and seafood (squid, shrimp, fish, and crab). Most pregnant women are prohibited from consuming fruits because of a belief that eating fruits can cause complications during delivery [4,5]. For example, 70% of 312 pregnant women in West Java Province are prohibited from consuming pomegranate and pineapple, believing that they may cause fetus abnormalities. Moreover, pregnant women often prefer consuming street food and fast food because of the taste, affordability, and satiety. Due to these factors, many women tend to ignore important nutrients during pregnancy and may have difficulty counting calories. Therefore, from 2018 to the present, the Indonesia Ministry of Health has implemented various direct and indirect interventions to address the causes of malnutrition during pregnancy through prenatal supplementary feeding programs [4]. However, the burden of malnutrition related to unhealthy and poor diets remains. Most women have misunderstandings about nutrition during pregnancy, which could exacerbate undernourishment and make it difficult to distinguish between adequate and excessive eating during pregnancy.

Introduced in 2017, the Nutrition Information System for Indonesian Pregnant Women (SISFORNUTRIMIL) is a potential web-based application prototype for recording and calculating food and calorie intake [6]. Women registered for this application can self-monitor dietary nutrition intake by selecting from five food choices, including beverages and snacks, at each mealtime. The food record tool has shown promise in improving healthy eating and helps health care professionals who have limited time to monitor the dietary intake of pregnant women [7]. Encouraging women to choose a variety of foods is one element that is required to facilitate the development of effective healthy eating that aims to alleviate nutrient deficiencies for both mothers and their children. This study aims to determine if the SISFORNUTRIMIL application is more effective than the traditional paper-based method for dietary self-monitoring. It was hypothesized that the application user group would achieve a calorie intake and maternal weight gain as recommended by guidelines compared with the nonuser group.

### Nutrition Information System for Pregnant Women in Indonesia

**Figure 1:**
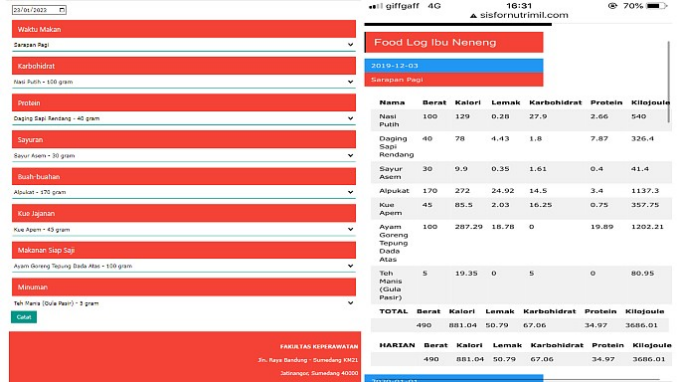
Screen capture of the "food record menu" of the Nutrition Information System for Indonesian Pregnant Women (SISFORNUTRIMIL) application.

Studies have tested if nutrition interventions among pregnant women enhance eating behavior, including food-based interventions using electronic mobile devices for dietary self-monitoring and weight management [6-8]. However, most studies of mobile apps only evaluate the tool’s utility rather than the intervention, so they do not comprehensively assess the mobile app’s impact [9]. In line with advancements in information and communication technology in Indonesia, internet users have increased by almost 10% in a year. Most people are connected to the internet and use mobile devices, including in rural areas [7]. This study focused on a dietary intake intervention using a web-based application. Pregnant women were instructed to use the food record tool to obtain the composite of food groups and balanced nutrients. The modified food record also enables assessment and calculation of calorie intake with the recommended amount for energy (2550 kcal), protein (97 g), fat (67.3 g), and carbohydrates (400 g) [10]. The recommended amounts are derived from Indonesian dietary guidelines for pregnant women and represent the principles of balanced nutrition through food diversity and safety, including the five food groups and portion size. The SISFORNUTRIMIL web-based application was created to integrate daily food recommendations and food records with automatic calorie estimation to prevent nutritional deficiencies during pregnancy (a screen capture of the "food record menu" of the SISFORNUTRIMIL application is shown in Figure 1). Hence, there is substantial potential to enhance awareness of a healthy and balanced diet and the daily requirements and recommendations for pregnant women. Besides, health care professionals can take advantage of the mobile app to convey knowledge and information, which may assist with behavior change [8].

Furthermore, based on existing studies, we believe that the SISFORNUTRIMIL application is a novel, alternative form of nutrition intervention to promote healthy eating among pregnant women. Indeed, it provides insight and contributes to the growing body of knowledge on maternal eating behaviors and positive pregnancy outcomes.

## Methods

### Study Design

We conducted a randomized controlled trial to examine the effectiveness of the SISFORNUTRIMIL application as an electronic food record tool, where users select from the food database system and include portion sizes and calories. The study was conducted between November 2019 and April 2020. This trial recruited pregnant women from 11 public maternal and child clinics across Bandung City, Indonesia. The participants were randomized into the intervention group (using the SISFORNUTRIMIL application for recording food) and the control group (using paper for recording food) in a simple randomization process (at a 1:1 ratio) using a web-based random number generator [11]. Allocated randomization began with a list of 56 randomly generated numbers between 1 and 112. The remaining numbers between 1 and 112 were allocated to the control group. Written informed consent was obtained from participants of this study.

**Figure 2:**
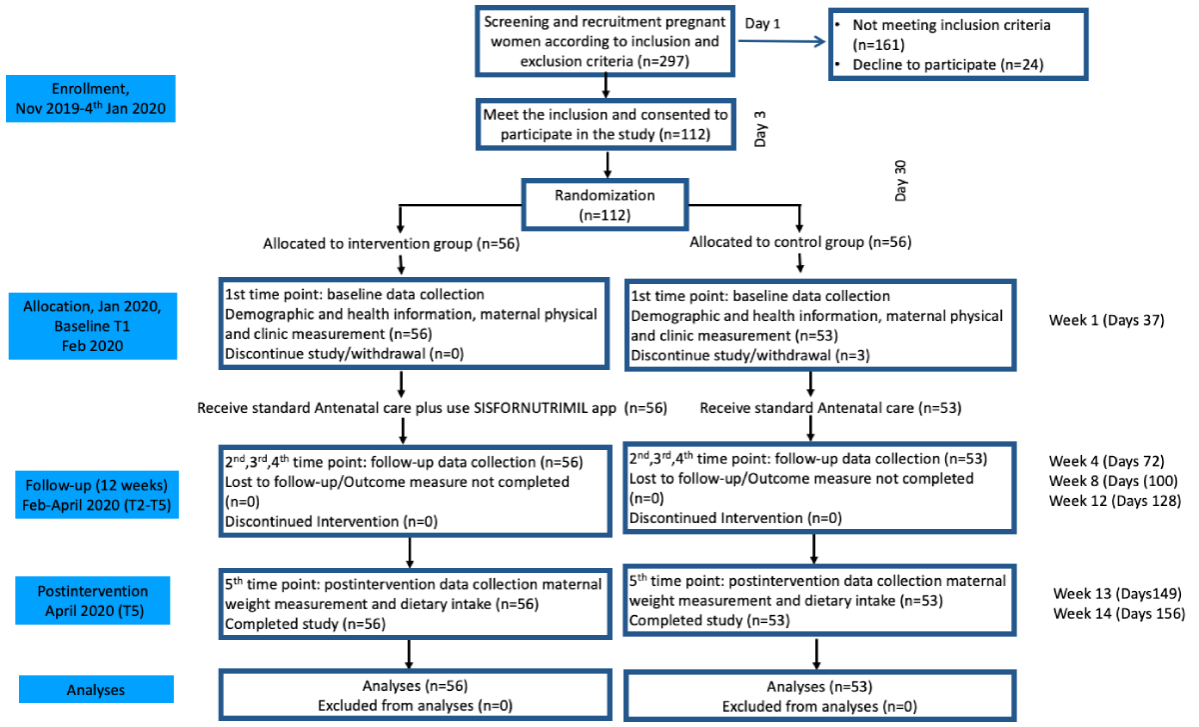
Participant flow diagram CONSORT (Consolidated Standards of Reporting Trials). SISFORNUTRIMIL: Nutrition Information System for Indonesian Pregnant Women.

The study was designed according to the recommendations of the CONSORT (Consolidated Standards of Reporting Trials) 2010 guidelines, in particular for nonpharmacological treatment [12]. The study flow chart is shown in Figure 2, and the detailed study protocol has been published previously [13].

### Participants

Recruitment commenced on November 3, 2019, and ended on January 4, 2020. Data collection began 1 week after each woman gave written informed consent. Participants were recruited from two sources: public health centers (PHCs) where pregnant women were registered and private practice clinics under the supervision of a PHC. All pregnant women who came for antenatal care and self-declared having difficulty counting calories were invited to the initial screening.

The researcher explained the study aims and study consent, and asked participants to complete the initial screening. We enrolled 298 pregnant women from 11 PHCs, including private clinics (Figure 2). All potential participants who returned the consent form were asked to complete a survey on demographic data and primary outcome measures; this survey was anonymous, and all information collected was confidential. Potential participants were eligible if they met the inclusion criteria and did not meet the exclusion criteria. The following inclusion criteria were used: permanent patients of the PHC, aged between 19-30 years, primigravida, gestational age 22-26 weeks, and currently using a mobile phone that has access to WhatsApp. Further, eligible participants were pair-matched according to maternal age, gestational age, parity, education level, and monthly household income, and were randomly assigned to the intervention and control groups. Patients were excluded during the enrollment process if they had a severe medical condition, history of miscarriage or stillbirth, or met the inclusion criteria but did not have the capacity to provide consent. The specific inclusion and exclusion criteria are summarized in Textbox 1. Those who met the criteria were scheduled for a face-to-face baseline measurement. A total of 3 women from the control group did not attend the baseline measurement, and a total of 109 participants were included at the baseline measurement, with slightly more participants in the intervention group (n=56) than the control group (n=53).

**Eligibility criteria.**

**Inclusion criteria**

- Permanent patient of public health center
- Maternal age between 19-30 years
- Primigravida and singleton pregnancy
- Gestational age 22-26 weeks
- Currently using a mobile phone with access to WhatsApp and the internet
- Systolic blood pressure at screening <140 mmHg and diastolic blood pressure <100 mmHg
- Normal BMI weight (kg/m^2^)
- Have monthly income
- Completed at least secondary education

**Exclusion criteria**

- Pregnant women with a severe medical condition such as food allergy, bulimia, and anemia
- Diagnosed with a mental illness patient or chronic illness
- History of miscarriage or stillbirth
- Participants who are unable to provide informed consent

### Description of Intervention

Both the intervention and control groups received standard antenatal care, including advice about healthy eating. Both interventions are rooted in the self-monitoring theory [14] and use the same research framework. Only the intervention group received standard prenatal care plus access to the SISFORNUTRIMIL application. All participants were asked to fill out food records 3 days a week on recommended days such as Monday, Friday, and one day on the weekend for 12 weeks (36 days in total). Additionally, on Sunday between 3 PM and 6 PM, both groups were sent a reminder to fill in the food record by the researcher through WhatsApp. Both groups also received a face-to-face baseline measurement and 3 follow-up measurements during anthropometric and clinical assessments. At the end of the intervention period, the participants from both groups completed the consumed dietary diversity survey [15]. The final clinical measurement for both groups was neonatal birth weight assessment.

### The Intervention Group

To access the menu in the system, users had to sign up and be verified by entering their personal information. The SISFORNUTRIMIL application is designed to access a network connection using HTTP; there is no need to install it, and it does not consume memory on the device.

After logging in, the women in this group could select and enter the food they had eaten in the food record tool. They could track their daily food and drink records, manage menu portions, and monitor calorie intake without counting manually. The system automatically saves data. This group’s expected change in eating behavior was for all users to follow a daily meal plan, read nutritional information, and review their intake in the food log to evaluate their caloric intake.

### The Control Group

The control group had no intervention after the baseline measurement but received standard prenatal care (nutrition counseling and pregnancy complication prevention), read the information sheet, and received nutrition guidelines and paper-based food records. They should have recorded their food intake for 12 weeks after baseline.

### Measurements

The dietary intake measures used were 3-day food records and the Minimum Dietary Diversity for Women (MDD-W) [15,16]. The dietary record for the intervention group was already created for the application, and this application enables its users to record all consumed food at each meal. The usability feature of the SISFORNUTRIMIL application provides food records tailored for Indonesian people. There are 321 types of food registered on the system, along with energy calorie amounts. The nutritional information of food items refers to the dietary guidelines from the Indonesian recommended dietary allowance (RDA) and estimated average requirements for pregnant women. Moreover, the maternal weight was measured using standard weight scales. During data collection, the researcher checked the weight scales regularly to ensure they were in good condition; if the scale cannot match the weight by adjusting the zero and span controls, the equipment should not be used.

### Sample Size and Power Calculation

This study estimated the sample size based on the result of the previous intervention study in Indonesia by Budiman et al [17] about “the correlation of the maternal factors with neonatal weight birth at PTH.” The initial sample size for each group (n=50) was computed as a function of a power level 1 – ß (.90), an α significance level (.05), and effect size *d* (0.66) by G*Power using an a priori power analysis and a *t* test of mean differences between the two independent means (two groups) based on the infant’s mean birth weight prediction (control: 2974.0 g, SD 417.2 g; intervention: 3250 g, SD 417.2 g). Assuming a 10% drop out, the sample size for each group should be 56, for a total of 112 participants.

### Statistical Analysis

Statistical analysis was carried out using SPSS 25 (IBM Corp), and the results are presented as means (SDs). Baseline and follow-up descriptive variables were compared across groups using univariate analyses. Independent *t* tests or Mann-Whitney *U* tests were used for nonparametric analysis. The mean of the change in score was calculated from baseline and follow-up measurements by assuming that the difference between both groups was 0.5.

### Ethics Approval

This randomized clinical trial was registered and published on ISRCTN (ISRCTN42690828). The research protocol was reviewed and approved by the Universitas Padjadjaran Research Ethics Committee on October 4, 2019 (reference 1227/UN6.KEP/EC/2019).

## Results

### Participants’ Baseline Characteristics

Of the 297 pregnant women recruited, only 112 met the inclusion criteria and were interested in participating in the study. A total of 186 pregnant women were excluded: 161 did not meet the inclusion criteria, and 25 people were considered suitable but declined to participate before randomization. In total, 112 pregnant women were randomly allocated into the intervention (n=56) and control groups (n=56). Only 109 participants conducted baseline measurements and completed the study (intervention: n=56; control: n=53; as seen in Figure 2). The mean age of all participants was 22.69 years. The most common mean gestational age of all participants was 23.97 weeks. Further participant demographics are provided in Table 1.

**Table 1:** Baseline characteristics between groups.

Description		Intervention group (n=56)	Control group (n=53)	*P* value	
**Characteristics, mean (SD)**					
	Maternal age (years)	22.77 (2.37)	22.62 (2.04)	.73	
	Gestational age (weeks)	23.98 (1.84)	23.96 (1.89)	.96	
	Maternal weight (kg)	54.75 (10.87)	54.68 (8.7)	.97	
**Educational and work history, n (%)**					.41
	No formal education	0 (0)	1 (2)		
	Primary school (9 years)	20 (36)	21 (40)		
	Secondary school (12 years)	32 (57)	24 (45)		
	Tertiary school (>12 years)	4 (7)	7 (13)		
**Occupation, n (%)**					.84
	Employment	18 (32)	18 (34)		
	Housewife	38 (68)	35 (66)		
**Total household income (IDR; US $), n (%)**					<.001
	<IDR 1.5 million (<99)	17 (30)	6 (11)		
	IDR 1.5-2 million (100-129)	37 (66)	46 (87)		
	>IDR 2 million (>$130)	2 (4)	1 (2)		
**Culture background, n (%)**					.58
	Sundanese	49 (88)	46 (87)		
	Javanese	7 (13)	6 (11)		
	Others	0 (0)	1 (2)		
**Daily language, n (%)**					.41
	Indonesia	26 (46)	28 (53)		
	Sundanese	51 (91)	42 (79)		
	Others	1 (2)	3 (6)		
**Avoid eating certain food, n (%)**					.40
	Yes	24 (43)	27 (51)		
	No	32 (57)	26 (49)		
**Avoid excessive weight gain, n (%)**					.11
	Yes	5 (9)	1 (2)		
	No	51 (91)	52 (98)		
**Avoid doing certain activities, n (%)**					.14
	Yes	12 (21)	18 (34)		
	No	44 (79)	35 (66)		
**Activity during pregnancy, n (%)**					.14
	Household chores/family care	42 (75)	52 (98)		
	Occupational activities	13 (23)	7 (32)		
	Sport and exercise	17 (30)	8 (15)		
**Take medication during pregnancy, n (%)**					.005
	Yes	40 (71)	49 (92)		
	No	16 (29)	4 (8)		
**Health problem before pregnancy, n (%)**					.91
	Yes	7 (13)	7 (13)		
	No	49 (88)	46 (87)		
**Morning sickness experience, n (%)**					<.001
	Yes	3 (5)	51 (96)		
	No	53 (95)	2 (4)		
**Seeking nutrition information, n (%)**					.03
	Yes	47 (84)	51 (96)		
	No	9 (16)	2 (4)		

### Differences in the Minimum Dietary Diversity Intake of Pregnant Women

After 12 weeks of the intervention, the minimum dietary diversity for reproductive age (MDD-W) had a significant difference (*P*=.005) between the intervention and control groups. The mean scores of MDD-W intake were 18.57 (SD 1.98) and 17.87 (SD 2.31) for the intervention and control groups, respectively (adjusted mean difference 0.77, 95% CI 0.28-1.25). A small effect size was found (*d*=0.28). Of the 10 MDD-W food groups, 8 showed a significant difference between the two groups. Most women in the intervention group showed improvements in their consumption of vegetables and fruits (mostly any dark green leafy vegetables and pulses, and vegetables rich in vitamin A) and animal sources (meat, poultry, fish, and eggs) than the control group (Table 2).

The staple and starchy food groups were consumed by 100% of the participants in the intervention and control groups. In this food group, the participants mostly consumed rice, porridge, noodles, and vermicelli. There was a trend in higher consumption of the following foods in the intervention group compared with the control group: other vegetables and tubers (adjusted mean difference 0.21 servings, 95% CI 0.07-0.36; *P*<.001; *d*=0.47), any dark green and leafy vegetables (adjusted mean difference 0.33 servings, 95% CI 0.19-0.47; *P*<.001; *d*=0.84), nuts and seeds (adjusted mean difference 0.27 servings, 95% CI 0.11-0.42; *P*<.001; *d*=0.67), and pulses group (adjusted mean difference 0.28 servings, 95% CI 0.12-0.44; *P*<.001; *d*=0.61). A small to large effect size was recorded.

There was also a significantly higher percentage of participants consuming animal sources: meat, poultry, and fish (adjusted mean difference 0.22 servings, 95% CI 0.05-0.40; *P*<.001; *d*=0.50), eggs (adjusted mean difference 0.14 servings, 95% CI –0.02 to 0.30; *P*<.001; *d*=0.33), vegetables and fruits rich in vitamin A (adjusted mean difference 0.16 servings, 95% CI 0.00-0.33; *P*<.001; *d*=0.46), and other fruits (adjusted mean difference 0.10 servings, 95% CI –0.06 to 0.27; *P*=.006; *d*=0.25). A small to medium effect size was recorded. In the milk and dairy group, the intervention group had a higher prevalence of consumption than the control group (mean 64.9% and 62.3%, respectively; adjusted mean difference 0.02 servings, 95% CI –0.15 to 0.20; *P*=.55; *d*=0.05).

The classification of 71 raw ingredients belonging to MDD-W’s 10 food groups was identified as having been consumed by the participants, as shown in Table 3. All food groups were observed in both groups. Food group 4 (other fruits) and food group 5 (other vegetables and tubers) were the most widely consumed groups, followed by food group 2 (other vitamin A–rich vegetables and fruits). The types of food derived from staple foods and starchy foods were also made into various dishes; white rice, porridge, fried rice, and crackers were frequently consumed by pregnant women. At the same time, the types of vegetables that are often cooked and consumed, such as water spinach/kangkong, turnip, and mushroom, are usually processed with food ingredients derived from processed nuts such as tofu, tempeh, and green beans.

**Table 2:** Differences in Minimum Dietary Diversity for Women (MDD-W) food groups consumed between the application user and nonuser groups.

			Baseline							Follow-up							*P* value			Effect size
			Intervention (n=56)		Control (n=53)		*P* value		Intervention (n=56)			Control (n=53)		Difference (95% CI)						
<5 food groups, n (%)			4 (7)		3 (6)		N/A^a^		0 (0)			2 (4)		N/A		N/A			N/A	
≥5 food groups, n (%)			52 (92)		50 (94)		N/A		56 (100)			51 (96)		N/A		N/A			N/A	
Diversity score, mean (SD)			6.61 (1.49)		6.64 (1.52)		.80		7.79 (1.20)			7.02 (1.39)		0.77 (0.28-1.25)		.005			0.28	
**Food groups of the MDD-W consumed (%), mean (SD)**																				
	Staple and starchy foods	100 (0)		100 (0)		>.99		100 (0)			100 (0)		0		>.99			0		
	Other vitamin A–rich vegetables and fruits	62 (14.21)		63 (10.2)		.19		82.1 (16.8)			65.4 (14.6)		0.16 (0.00 to 0.33)		<.001			0.46		
	Any dark green and leafy vegetables	70 (26.36)		73 (20.78)		.25		99.4 (4.5)			66 (22.2)		0.33 (0.19 to 0.47)		<.001			0.84		
	Other fruits	62 (11.64)		60.69 (13.8)		.29		77.4 (20.2)			66.7 (19.6)		0.10 (–0.06 to 0.27)		.006			0.25		
	Other vegetables and tubers	73 (26.78)		72.62 (28.86)		.47		92.3 (18)			70.4 (22.3)		0.21 (0.07 to 0.36)		<.001			0.47		
	Meat, poultry, and fish	57.70 (16.2)		58.06 (16.02)		.45		79.8 (20.8)			57.2 (17.8)		0.22 (0.05 to 0.40)		<.001			0.50		
	Eggs	58.10 (16.4)		57.48 (15.9)		.42		81 (17.8)			66.7 (22.6)		0.14 (–0.02 to 0.30)		<.001			0.33		
	Pulses (beans, peas, and lentils)	57.70 (17.7)		58.68 (16.11)		.38		90.5 (16.5)			61.6 (20)		0.28 (0.12 to 0.44)		<.001			0.61		
	Nuts and seeds	56.55 (12.4)		55.43 (10.89)		.30		89.9 (16.7)			62.9 (12.5)		0.27 (0.11 to 0.42)		<.001			0.67		
	Milk and dairy	52.72 (31.3)		54.72 (32.28)		.37		64.9 (22.4)			62.3 (23.6)		0.02 (–0.15 to 0.20)		.55			0.05		

^a^N/A: not applicable.

**Table 3:** Food items observed in pregnant women’s food intake by food groups.

Food groups of the MDD-W^a^	Food items, n	Items consumed by the participants
Staple and starchy foods	8	Rice, porridge, bread, noodle, rice cake, cereal
Other vitamin A–rich vegetables and fruits	9	Carrots, pumpkin, sweet potatoes, jack fruits, mango, papaya
Any dark green and leafy vegetables	5	Spinach, water spinach (kangkong)
Other fruits	14	Sweet banana, orange, apple, kiwi, strawberry, watermelon, avocado, snake skin’s fruits, dragon fruits, grapes, melon
Other vegetables and tubers	16	Broccoli, cassava leaves, lettuce, sprouts, cabbage, cucumber, tomato, eggplant, onion, bitter melon, cauliflower, green beans, beans, white potatoes, cassava, taro
Meat, poultry, and fish	6	Chicken, beef, dried fish, fish, lamb
Eggs	2	Eggs from chicken
Pulses (beans, peas, and lentils)	5	Tofu, tempeh, green beans
Nuts and seeds	2	Peanut, chia
Milk and dairy	4	Milk, yogurt, cheese

^a^MDD-W: Minimum Dietary Diversity for Women.

### Differences in the Dietary Intake of Pregnant Women

Participants’ dietary intake in both groups was reported for 3 days for 12 weeks. All participants’ follow-up food records were subsequently recorded for further analysis. For this analysis, we used average food group intakes, referring to the Indonesian RDA.

Table 4 summarizes the energy intake in kcal and the distribution of essential nutrients per day between the intervention and the control groups. The mean energy kcal intake in the intervention and control groups was significantly different (*P*=.02)*.* All pregnant women in the intervention group showed adequate energy intake (mean 2502.46, SD 111.6 kcal); this amount is in accordance with the recommended serving for the third trimester of pregnancy (2550 kcal per day). The mean energy intake in the control group did not meet the recommendation (mean 2659.34, SD 113.99 kcal). The difference in energy intake between the intervention group and the control groups was 156.88 kcal (95% CI 114.52-199.23; *d*=–1.39). A large effect size was found. Additionally, the daily intake was significantly different between the intervention and control groups (*P*=.001). The macro- and micronutrients in the intervention group demonstrated an adequate intake of carbohydrates (mean 304.15, SD 56.65), protein (mean 70.17, SD 8.61), fat (mean 60.13, SD 6.56), iron (mean 27.44, SD 6.03), and vitamin C (mean 77.83, SD 9.24). Additionally, the macro- and micronutrient intake in the control group did not meet recommendations for protein (mean 55.84, SD 6.94), iron (mean 20.5, SD 4.44), and vitamin C (mean 55.36, SD 70.12), and exceeded recommendations for carbohydrates (mean 406.58, SD 60.48) and fat (mean 71.09, SD 8.00).

**Table 4:** Differences in energy, vitamins, and minerals between applications user and nonuser groups within 12 weeks.

Variable	Intervention (n=56)			Control (n=53)				Differences (95% CI)		*P* value^a^		Effect size
	Baseline	Follow-up	RDA^b^ (%)	Baseline	Follow-up	RDA (%)						
Energy (kcal), mean (SD)	2361.07 (163.54)	2502.46 (111.6)	98.13	2236.96 (275.37)	2659.34 (113.99)	104.28	156.88 (114.5 to 199.23)		.002		–1.39	
Protein (g), mean (SD)	52.58 (10.83)	70.17 (8.61)	92.10	55.47 (7.94)	55.84 (6.94)	73.47	14.33 (11.40 to 17.25)		<.001		1.83	
Fat (g), mean (SD)	69.83 (7.22)	60.13 (6.56)	70.74	70.28 (30.83)	71.09 (8)	106.11	–10.96 (–13.71 to –8.20)		<.001		–1.49	
Carbohydrate (g), mean (SD)	318.46 (50.6)	304.15 (56.65)	87.14	327.4 (60.48)	406.58 (64.42)	116.49	–102.43 (–125.2 to –79.60)		.02		–1.68	
Iron (mg), mean (SD)	20.54 (4.22)	27.44 (6.03)	70.35	20.41 (4.63)	20.5 (4.4)	52.33	6.94 (4.96 to 8.91)		.008		1.31	
Vitamin C (mg), mean (SD)	66.46 (7.07)	77.83 (9.24)	78.18	60.19 (15.48)	55.36 (70.12)	65.12	22.47 (3.43 to 41.50)		.03		1.38	

^a^*P* value obtained from the independent sample *t* test.

^b^RDA: recommended daily allowance.

### Differences in the Maternal Weight Gain of Pregnant Women

As shown in Table 5, there was a significant difference in maternal weight gain between the intervention and control groups (*P*<.001). After the intervention, the maternal weight gain in the intervention group slightly increased by 6.21 kg (mean 60.96 kg, SD 10.47 kg), following the recommendations of the Institute of Medicine (IOM). In contrast, women’s weight in the control group increased by 7.79 kg (mean 62.47 kg, SD 11.16 kg) from the baseline measurement, exceeding the recommended standard. In other words, the recommended gain for the rest of the second and third trimesters was 0.5 kg per week (Table 5).

**Table 5:** Differences in maternal weight measurement between application user and nonuser groups within and after 12 weeks.

Variable	Intervention group maternal weight (kg; n=56), mean (SD)	Control group maternal weight (kg; n=53), mean (SD)	Effect size	*P* value^a^
Baseline	54.75 (10.87)	5354.68 (8.7)	N/A^b^	N/A
Change in 4 weeks	56.8 (10.26)	57.26 (9.71)	N/A	N/A
Change in 8 weeks	58.83 (10.13)	58.83 (10.13)	N/A	N/A
Change in 12 weeks	60.96 (10.47)	62.47 (11.16)	0.05	.97
*P* value^c^	<.001	<.001	N/A	N/A

^a^*P* value was analyzed using Mann-Whitney *U* test.

^b^N/A: not applicable.

^c^*P* value was analyzed using Friedman test.

## Discussion

### Principal Findings

To our knowledge, this is the first study of its kind in Indonesia. Moreover, the number of randomized controlled trial studies worldwide to evaluate mobile apps’ impact on monitoring dietary change and body weight is limited. This study focuses on an intervention, regular self-monitoring of dietary intake for healthy weight gain during pregnancy by using a food record in the SISFORNUTRIMIL application. The findings of our study indicate that the SISFORNUTRIMIL application was more effective than the traditional paper-based method in improving a balanced diet and positive weight gain during pregnancy; in addition, we reported that the amount of calorie intake and maternal weight among the application users was relatively achieved according to the recommended guidelines compared to the application nonuser group.

Similar to a previous study [18], we found that mobile technology apps are a potential intervention in nutrition self-monitoring practices to improve maternal nutrition during pregnancy. Most studies were focused on weight loss for people with overweight [19,20]. For instance, a study in North Carolina (Durham, Orange, and Wake) showed that smartphone apps are relevant for weight management and food tracking [21]. In this study, the findings showed that the women in the intervention group who received food suggestions and recorded their intake through the SISFORNUTRIMIL application consumed the 10 MDD-W food groups significantly more than the women who used the paper-based method only (control group). Additionally, the dietary intake of energy and energy-yielding nutrients, including carbohydrates, protein, fat, and micronutrients (iron and vitamin C) for most women in the intervention group was in accordance with the dietary recommendations. These food groups were noted as other vitamin A–rich vegetables and fruits, any dark green leafy vegetables, other fruits, vegetables and tubers, pulses, nuts, and animal sources (meat, poultry, fish, and eggs), which provide more varying amounts of micronutrients.

The 10 MDD-W food groups have been used to identify women’s food security as well as be an indicator of access to food [22]. The diets of all women in both groups were dominated by rice and starchy staples. Rice is the most consumed food by most ethnic groups in Indonesia, including ethnic Sundanese in West Java [23]. Specifically, most of the 5 food groups consumed by women in the intervention group were higher than the control group. The following are the mean percent of consumed food in each MDD-W food group in the intervention and control groups: other vitamin A–rich vegetables and fruits (mean 82.1%, SD 16.8% vs mean 65.4%, SD 14.6%), dark green leafy vegetables (mean 99.4%, SD 4.5% vs mean 66%, SD 22.2), other vegetables and tubers (mean 92.3%, SD 18% vs mean 70.4%, SD 22.3%), eggs (mean 81%, SD 17.8% vs mean 66.7%, SD 22.6%), pulses (mean 90.5%, SD 16.5% vs mean 61.6%, SD 20%), and nuts (mean 89.9%, SD 16.7% vs mean 62.9%, SD 12.5%). Women in the control group had a limited intake of nutrients from consuming fruits and vegetables. Additionally, eggs; pulses; nuts and seeds; and foods rich in protein, vitamin B, and saturated fatty acids were rarely consumed. Consuming a more diverse diet is beyond the reach of most women in the control group (application nonusers) because of inconsistency in consuming food regularly. It has been proven through food records that infrequent meals and reduced portion sizes could lead to low dietary diversity. This was confirmed in this study; most pregnant women who received food choices and monitored their intake through food records in the application successfully achieved a more balanced diet.

The SISFORNUTRIMIL application was designed as a self-monitor dietary intake tool that includes all food items within the food composition database and their macronutrient recommendations. Most pregnant women in the intervention group met the daily servings of energy calorie intake in accordance with the RDAs for Indonesian pregnant women, while the energy intake of women in the control group exceeded the RDA targets. The mean intake of carbohydrates, protein, and fat followed the dietary recommendations; additionally, the intake of iron and vitamin C met the recommendation [24]. Several studies have stated that energy intake requirements in pregnancy are not aimed at weight maintenance but at appropriate rates of weight gain, which in turn, minimizes the risks of adverse outcomes in the mother and her offspring [25]. In other words, energy is the primary nutrient determinant of gestational weight gain (GWG), although specific nutrient deficiencies may restrict that gain [26]. Further, a few studies have found that inadequate iron intake is associated with insufficient red blood cells and leads to iron deficiency anemia [27]. About 25% of all maternal deaths globally are associated with severe anemia iron deficiency and a significant risk for maternal bleeding, premature birth, and having a baby with low birth weight [28].

This study found the mean maternal weight gain of women in the intervention group at the end of pregnancy was consistent with the IOM recommendations, in which the IOM guideline established a cutoff point for weight gain for each trimester according to the normal BMI for pregnant women (0.31-0.65 kg/week) [29]. Increases were seen from a mean of 54.75 kg to 60.96 kg (difference 6.21 kg) in the intervention group, while the participants in the control group increased from a mean of 54.68 kg to 62.47 kg (difference 7.79 kg). There could be a relevant association between dietary intake within 12 weeks of the intervention and GWG, which was not included in the analysis. A previous study in Michigan reported that energy intake was associated with increased GWG [30]. Another study by Hrolfsdottir et al [31] indicated that balanced energy/protein supplementation was associated with a slight increase in GWG. In contrast, Kominiarek and Peaceman [32] assumed that weight gain depended on parity, whereas the weight gain in multigravida women was lower than in primigravida women. However, this is not evident when adjusting for energy intake, which does not change during pregnancy [33].

Our study has numerous strengths, including the recruitment of participants using inclusion criteria and the feasibility randomized control method with a control group to allow for adequate testing. Furthermore, the SISFORNUTRIMIL application provides a platform to help pregnant women with healthy dietary practices throughout pregnancy. Pregnant women who regularly monitor their intake on food records tend to have a high-quality diet, which can cause positive pregnancy outcomes. Some studies claim that improving the quality of women’s diet and decreasing preventable pregnancy complications are associated with the achievement of a nutrition intervention [34-37]. Therefore, even though the effectiveness of the SISFORNUTRIMIL application focuses on increasing dietary intake, the study still had a nonspecific effect on healthy maternal weight. Additionally, concerning reducing stunting prevalence, this intervention shows that the SISFORNUTRIMIL application had a significant effect on maternal diet and the potential to give birth to healthier babies. Thus, taken together, our findings indicate that the SISFORNUTRIMIL application could play an important role in health dynamics in Indonesia.

Considering the inadequate nutrition in Indonesia, the SISFORNUTRIMIL application could be used as an alternative tool for self-dietary assessment in improving healthier dietary practices during pregnancy. Importantly, the pregnant women who used this application consumed more of the five food groups and achieved a more balanced diet, highlighting the difficulty in estimating calorie intake. Furthermore, health professionals, including doctors, midwives, and nutritionists, can consider using this application to overcome the challenges of limited counseling time and human resources. The application may also support the Indonesian government’s strategy of reducing maternal and child mortality, eliminating all forms of hunger, and reducing intrauterine growth stunting by 2023.

Further research is necessary to examine the effectiveness of the SISFORNUTRIMIL application on the mother’s clinical condition, the neonatal birth weight, and the users’ experience in consistently inputting dietary data. These will be important in the long-term maintenance of changes to dietary behavior.

### Limitations

A limitation of this study is the small sample size and short duration, which are likely to be at risk of bias, so these results have to be interpreted with caution. As a result, the interpretation of the findings has also focused on more recent studies that can be considered of higher quality. In future research, there is a need to use relatively large sample sizes, long durations, and innovative approaches.

### Conclusions

The SISFORNUTRIMIL application intervention had both direct and indirect impacts to support various components of maternal and child health services. It also may directly impact the efficiency of nutrition intervention costs in LMIC. Our findings do not support monitoring other micronutrients, and this should be developed in future work, including a widespread assessment of the impact of biomarker nutrients. Policy makers and nutritionist program personnel should use this to increase maternal and child health practices.

## Appendix

Multimedia Appendix 1 CONSORT checklist.

## Abbreviations

CONSORT: Consolidated Standards of Reporting Trials
GWG: gestational weight gain
IOM: Institute of Medicine
LMIC: low- and middle-income countries
MDD-W: Minimum Dietary Diversity for Women
PHC: public health center
RDA: recommended dietary allowance
SISFORNUTRIMIL: Nutrition Information System for Indonesian Pregnant Women

## Data Availability

Data are available upon reasonable request.
